# Investigating gateway effects using the PATH study

**DOI:** 10.12688/f1000research.18354.2

**Published:** 2019-12-04

**Authors:** Peter Lee, John Fry

**Affiliations:** 1 P.N.Lee Statistics and Computing Ltd, Sutton, Surrey, SM2 5DA, UK; 2 RoeLee Statistics Ltd, Sutton, Surrey, SM2 5DA, UK

**Keywords:** Cigarettes, Confounding, E-cigarettes, Gateway effects, Modelling, Propensity score

## Abstract

**Background**: A recent meta-analysis of nine cohort studies in youths reported that baseline ever e-cigarette use strongly predicted cigarette smoking initiation in the next 6-18 months, with an adjusted odds ratio (OR) of 3.62 (95% confidence interval 2.42-5.41). A recent e-cigarette review agreed there was substantial evidence for this “gateway effect”. As the number of confounders considered in the studies was limited we investigated whether the effect might have resulted from inadequate adjustment, using Waves 1 and 2 of the US PATH study.

**Methods**: Our main analyses considered Wave 1 never cigarette smokers who, at Wave 2, had data on smoking initiation.We constructed a propensity score for ever e-cigarette use from Wave 1 variables, using this to predict ever cigarette smoking. Sensitivity analyses accounted for other tobacco product use, linked current e-cigarette use to subsequent current smoking, or used propensity scores for ever smoking or ever tobacco product use as predictors. We also considered predictors using data from both waves, attempting to reduce residual confounding from misclassified responses.

**Results**: Adjustment for propensity dramatically reduced the unadjusted OR of 5.70 (4.33-7.50) to 2.48 (1.85-3.31), 2.47 (1.79-3.42) or 1.85 (1.35-2.53), whether adjustment was made as quintiles, as a continuous variable or for the individual variables. Additional adjustment for other tobacco products reduced this last OR to 1.59 (1.14-2.20). Sensitivity analyses confirmed adjustment removed most of the gateway effect. Control for residual confounding also reduced the association.

**Conclusions**: We found that confounding is a major factor, explaining most of the observed gateway effect. However, our analyses are limited by small numbers of new smokers considered and the possibility of over-adjustment if taking up e-cigarettes affects some predictor variables. Further analyses are intended using Wave 3 data to try to minimize these problems, and clarify the extent of any true gateway effect.

## Introduction

In youths, use of e-cigarettes (“vaping”) and cigarette smoking are strongly associated, as shown in, e.g. Canada ( Aleyan *et al*., 2018), France ( Dautzenberg *et al*., 2016), Great Britain ( Eastwood *et al*., 2015), Korea ( Lee *et al*., 2014) and Poland ( Goniewicz *et al*., 2014), as well as the U.S. (e.g. Cooper *et al*., 2016; Dutra & Glantz, 2014; Wills *et al*., 2017). Since vaping significantly reduces exposure to harmful constituents compared to smoking ( National Academies of Sciences Engineering and Medicine, 2018), one might expect risks from vaping to be substantially lower ( Nutt *et al*., 2014). While the benefits of introducing e-cigarettes would seem clear for smokers switching to e-cigarettes who would have continued smoking otherwise, for established smokers who are helped to quit, and for individuals who would otherwise have started smoking who start vaping instead, there are possible downsides. While risk increases may be modest for smokers intending to quit who vape instead, and for smokers who vape but retain their usual cigarette consumption, there is concern that vaping may encourage youths to start smoking who would otherwise not have done so, this possibility being the focus of our paper.

There are two potential contributors to any observed association between vaping and the subsequent initiation of smoking. One is “common liability”, with youths who choose to vape already possessing attributes which make them more likely to smoke, and the other is a true causal effect of vaping, the so-called “gateway effect”. Obtaining evidence to determine the extent to which any observed association is actually due to a true gateway effect, and not confounded by common liability is not straightforward, and may need to be addressed not only, as here, by detailed study of data from a prospective cohort study, but by looking at trends in smoking prevalence in countries where vaping has increased ( Etter, 2018; Lee *et al.*, 2018).

Recent analyses ( Soneji *et al*., 2017) based on nine U.S. cohort studies in young people have linked previous vaping to subsequent initiation of smoking. This publication reported that among baseline never-smokers, ever vaping at baseline strongly predicted initiation in the next six to 18 months, with an odds ratio (OR) of 3.62 (95% confidence interval (CI) 2.42-5.41) after adjustment for various predictors of initiation. Similarly baseline past 30-day vaping also predicted subsequent 30-day cigarette use (OR 4.25, 95% CI 2.52-7.37).

A recent review of e-cigarettes ( National Academies of Sciences Engineering and Medicine, 2018) considered this to provide “substantial evidence” of a gateway effect, noting the “wide range of covariates” that the relevant studies had considered, and thought it “unlikely” that confounding entirely accounts for the association, as reductions in the association following adjustment were not consistently observed.

While some studies ( Barrington-Trimis *et al*., 2016; Primack *et al*., 2015; Primack *et al*., 2016) do report that the association increases following adjustment, many more ( Best *et al*., 2018; Conner *et al*., 2018; Hammond *et al*., 2017; Hornik *et al*., 2016; Leventhal *et al*., 2015; Loukas *et al*., 2018; Lozano *et al*., 2017; Miech *et al*., 2017; Spindle *et al*., 2017; Unger *et al*., 2016; Watkins *et al*., 2018; Wills *et al*., 2017) report a decrease. Furthermore, though adjusted associations are usually statistically significant, adjustment is often limited. Factors never considered include, for example, school performance, parental smoking and peer attitudes to smoking. In order to gain better insight into the magnitude of any true gateway effect, information from a large cohort study which collected data on very many factors would therefore clearly be useful, as would gaining some insight into the extent of bias resulting from misclassification of such variables.

Here we report detailed analyses of the gateway effect based on Wave 1 (2013–2014) and Wave 2 (2014–2015) of the Population Assessment of Tobacco and Health (PATH) study ( Berry *et al*., 2019b; Hyland *et al*., 2017), a longitudinal cohort study in the U.S. supported by federal funds. The databases provide extensive information on tobacco product use and on many other factors possibly linked to smoking initiation. At each Wave, data are separately collected for youths aged 12–17 and adults aged 18+, and our analyses, which concern smoking initiation by youths, use the youth data of each Wave, plus Wave 2 data for adults previously youths at Wave 1.

## Methods

### The main analysis

The main analysis considers those who had never smoked cigarettes by Wave 1 and who, at Wave 2, had information available on whether initiation of cigarette smoking had occurred. Use of other tobacco products is not considered. For a youth to be considered, data should be available on each of five demographics (age, sex, Hispanic origin, race, region) and on vaping.

The analyses, which relate ever having vaped by Wave 1 to initiation of cigarette smoking by Wave 2, after adjustment for factors linked to e-cigarette use recorded at Wave 1, was conducted in two steps.

***Step 1.*** In step 1, Wave 1 data were used to develop a propensity score for e-cigarette use based on the five demographic variables and on 60 smoking predictor variables selected from a much longer list. As described more fully in the Extended Data, we ignored questions only asked in a population subset, only really relevant to smokers, or of dubious relevance. Also, where many related questions were asked, attention was limited to those seemingly more likely to be relevant.

We used a logistic regression model where the propensity for vaping ( *P _i_*) for a youth *i* ( *i* = 1 to n) was linked to various smoking predictors *x _ij_* ( *j =* 1 to m) ** by

$${log}_{e}\left(\frac{P_{i}}{1-P_{i}}\right)=a_{0}+\sum_{j=1}^{m}a_{j}x_{ij}$$

For a given set of predictors, we refer to the value of the term on the left as the propensity score.

All logistic regression analyses were weighted by the person-level weights provided on the PATH database, with the weights normalized to sum to 1.

Introducing all 65 variables simultaneously into the model would have involved two problems. First, as the analysis required individuals with complete data on all variables, substantial information may be lost. Second, analyses including very many variables sometimes fail to solve. We therefore introduced variables in stages, using groups of conceptually-related variables, with missing values likely to be on the same individuals.

At stage 1 the variables were divided into groups numbered 1–11. In each group, an analysis was carried out for each variable individually, followed by a forward stepwise approach with the most significant variable introduced first, then the next, until no further variables in the group significant at p<0.01 could be introduced. At stage 2 significant variables from the first stage were divided into three groups (A, B and C), and a forward stepwise approach again used to identify significant variables.

Finally, at stage 3, the stepwise approach was applied to the stage 2 significant variables to generate a final list of variables for the propensity score, which was then re-calculated based on youths with complete data on all these variables.

At each stage, the analyses involved all participants with complete data for each variable considered in the group being analysed.

***Step 2.*** Step 2 involved the outcome of interest, initiation of cigarette smoking between Wave 1 and Wave 2. The first analysis was a weighted logistic regression analysis to determine the unadjusted OR and 95% CI for the association of ever vaping at Wave 1 with subsequent initiation of cigarette smoking. The second analysis was similar but adjusted for propensity by dividing the youths into five quintiles of the propensity score, the separate OR (95% CI) values for each stratum being then combined to form an overall propensity-adjusted estimate. Propensity-adjusted analyses were also conducted using the score as a continuous variable, and also using the variables making up the score individually rather than combined.

### Sensitivity analyses

Five sets of sensitivity analyses were conducted linking vaping to initiation of cigarette smoking, along the lines of the main analysis. For each set, ORs (95% CIs) were again calculated with no adjustment for propensity, adjustment as quintiles, adjustment as a continuous variable, and adjustment for the variables making up the score.

**Sensitivity analysis 1** Youths who, by Wave 1, had ever used any other tobacco product (i.e. than cigarettes or e-cigarettes) were excluded. As there were considerably fewer significant predictor variables from the stage 1 analyses, the stage 2 analyses were omitted.

**Sensitivity analysis 2** Here ever users of other tobacco products by Wave 1 were not excluded but use of other products was included as an extra predictor. The stage 1 and 2 analyses were not repeated. Rather the final model used was one that included the same final set of variables plus that for ever using other tobacco products.

**Sensitivity analysis 3** Whereas the main analysis and sensitivity analyses 1 and 2 linked a propensity score for ever vaping by Wave 1 to ever cigarette smoking by Wave 2, sensitivity analysis 3 linked a propensity score for current e-cigarette use at Wave 1 to current cigarette smoking at Wave 2, last 30-day use being considered current. Again, as few significant variables emanated from the stage 1 analyses, the stage 2 analyses were omitted.

**Sensitivity analysis 4** Whereas all the analyses described above relate to a propensity score for vaping, sensitivity analysis 4 was essentially the same as for the main analysis but based on a propensity score for ever cigarette smoking.

**Sensitivity analysis 5** Sensitivity analysis 5 was also like the main analysis, but the propensity score was based on ever use of any tobacco product.

For sensitivity analyses 4 and 5, the full three-stage process described in Step 1 was used to determine the variables included in the propensity score.

### Analyses investigating residual confounding

In the main analysis, the propensity score for e-cigarettes used data provided by youths at Wave 1. Although there was no gold standard to validate reported answers, it seemed possible that more accurate predictors could be based on data from Wave 1 and 2 combined. For those variables forming the propensity score we investigated whether there was further useful information available in Wave 2 and, if so, created a revised variable. How this was done is detailed in the Results section, the procedure depending on which variables were selected for the propensity score. Once the revised predictor variables were created, the main analyses were rerun using a modified propensity score.

### Software

Relevant data were transferred for analysis to a ROELEE database, and analysed using the ROELEE program (Release 59, Build 49). SAS version 9.4 (SAS Institute Inc, 2017) was also used to check some results from ROELEE and generate results when ROELEE failed to converge. The GLM Package and the Step function from the R Program ( https://www.r-project.org) could be used to run all the analyses.

## Results

### Main analysis

The propensity score for ever vaping by Wave 1 was developed using five demographic variables and 60 other predictors ( Table 1). Each variable, except for variable 52, depended on a question involving a few possible answers (see Table 1 footnotes) with the regression analyses estimating a coefficient per level. Exceptionally, variables 10–13, where numbers of youths were very small for some levels, were treated as continuous variables in analysis.

**Table 1.** Wave 1 predictor variables used with details of which stage they were eliminated from consideration. For each of the 65 Wave 1 predictor variables considered, Table 1 gives details of their type (graded or continuous) and of the stage at which they were eliminated from consideration for the final propensity score.

Group		Variables	Levels or continuous ^a^	Eliminated at stage 1 ^b^	Eliminated at stage 2 ^c^	Eliminated at stage 3 ^d^
1		**Demographic variables**				
	1	Age range	2	No → A	No → F	No → P
	2	Gender	2	No → A	No → F	No → P
	3	Hispanic origin	2	Yes		
	4	Race	3	Yes		
	5	Census region	4	Yes		
2		**Education, internalizing disorders**				
	6	Highest grade or year at school completed by parent	5	Yes		
		Last time significant problems with:				
	7	(a) Feeling very trapped, lonely, sad, blue, depression	4	No → A	No → F	Yes
	8	(b) Sleep trouble – such as bad dreams, sleeping restlessly	4	Yes		
	9	(c) Becoming very distressed when something reminded of past	4	No → A	Yes	
3		**Susceptibility to smoking**				
	10	Ever been curious about smoking a cigarette	4	No → B	No → F	No → P
	11	Think you will smoke a cigarette in the next year	4	No → B	No → F	No → P
	12	Think you will try a cigarette soon	4	Yes		
	13	Would smoke a cigarette if one your best friends offered you one	4	Yes		
4		**Externalizing disorders**				
		Last time 2+ times				
	14	(a) Lied or conned to get things you wanted or avoid doing something	4	No → B	Yes	
	15	(b) Had a hard time paying attention at school, work or home	4	No → B	Yes	
	16	(c) Had a hard time listening to instructions at school, work or home	4	Yes		
	17	(d) Were a bully or threatened other people	4	Yes		
	18	(e) Started physical fights with other people	4	No → B	Yes	
	19	(f) Felt restless or the need to run around or climb things	4	Yes		
	20	(g) Gave answers before the other person finished asking the question	4	Yes		
5		**Attitudes to tobacco**				
		Agree/disagree				
	21	(a) I think I would enjoy using tobacco	4	No → B	No → F	No → P
	22	(b) Using tobacco would be energizing	4	Yes		
	23	(c) Using tobacco would help me reduce or handle stress	4	No → B	Yes	
	24	(d) Using tobacco would help me calm down when I am angry	4	Yes		
	25	(e) Using tobacco would help me control my weight	4	Yes		
	26	(f) Using tobacco would help me feel more comfortable at parties	4	Yes		
	27	How much you think people harm themselves when they smoke cigarettes every day	4	Yes		
	28	How much you think people harm themselves when they smoke cigarettes some but not every day	4	Yes		
	29	How long do you think someone has to smoke cigarettes before it harms their health	6	Yes		
	30	Agree/disagree: some tobacco products are safer than others	5	No → B	No → F	No → P
	31	Seen a tobacco sweepstakes ad in past 6 months	2	Yes		
6		**Substance use**				
	32	Ever used alcohol at all	2	No → B	No → F	No → P
	33	Ever used prescription drug not prescribed to you: Ritalin or Adderall	2	No → B	No → F	No → P
	34	Ever used prescription drug not prescribed to you: Painkillers, sedatives or tranquilizers	2	Yes		
	35	Ever used substance: Cocaine or crack	2	Yes		
	36	Ever used substance: Stimulants like methamphetamine or speed	2	Yes		
	37	Ever used substance: Any other drugs like heroin, inhalants, solvents or hallucinogens	2	Yes		
7		**Risk taking variables**				
		Agree/disagree:				
	38	(a) Like to do frightening things	5	Yes		
	39	(b) Like new and exciting experiences even if I have to break the rules	5	No → C	No → F	Yes
	40	(c) Prefer friends who are exciting and unpredictable	5	No → C	No → F	No → P
8		**School performance, Health Behaviour** **Overall, Anxiety**				
	41	Youth’s grade performance in school in past 12 months	10	No → C	No → F	Yes
	42	How often youth missed school due to illness in past 12 months	5	Yes		
	43	Youth’s overall health status (as reported by parent or guardian)	5	Yes		
	44	Last time problems with: feeling very anxious, nervous, tense, scared, panicked	4	No → C	Yes	
9		**Accessibility to tobacco,** **Susceptibility to social influences**				
	45	How easy you think it is for people your age to buy tobacco products in a store	4	No → C	Yes	
	46	Hours spent watching TV on a typical day	5	Yes		
	47	How often do you use the internet	7	Yes		
	48	Has a Facebook, Google Plus, MySpace, Twitter or other social networking	2	No → C	No → F	No → P
10		**Family-related variables**				
	49	Parent has a spouse or partner that lives in household	2	Yes		
	50	Cigarettes or tobacco might be available to youth at parent or guardian’s home	2	Yes		
	51	Anyone who lives with you now uses tobacco	3	No → C	No → F	No → P
	52	Number of hours in past 7 days you were in close contact with others when they were smoking	C	Yes		
	53	Parent or guardian marital status	3	Yes		
	54	Youth has a curfew or set time to be home on school nights	2	Yes		
	55	Youth has a curfew or set time to be home on weekend nights	2	Yes		
	56	Rules about using tobacco products that are burned inside home	3	Yes		
	57	Statement that best describes the rules about using combustible tobacco	3	No → C	Yes	
	58	Parent or guardian talk with you, even once about not using any type of tobacco	2	Yes		
	59	Reaction of parent/guardian found you using tobacco	3	No → C	No → F	No → P
11		**Pocket money allowance, Movie** **influence**				
	60	Money received in total during an average week	9	No → A	No → F	Yes
	61	Number of times seen Movie 1	4	Yes		
	62	Number of times seen Movie 2	4	No → A	No → F	Yes
	63	Number of times seen Movie 3	4	No → A	Yes	
	64	Number of times seen Movie 4	4	No → A	No → F	Yes
	65	Number of times seen Movie 5	4	Yes		

^a^For graded variables, the number of levels is shown in the table, and the levels they represent is shown below. Continuous variables are indicated by the letter C. Exceptionally variables 10–13 were treated as a continuous variable with levels 1 to 4. ^b ^Those not eliminated at stage 1 went into stage 2 analyses A, B or C as indicated. ^c^Those variables not eliminated at stage 2 went into stage 3 (final = F) analysis. ^d^Those variables not eliminated at stage 3 were included in the propensity score (P).

**Table:** 

Grading systems used:	Variable(s)
12–14; 15–17	1
Male; Female	2
Hispanic; Non-Hispanic	3
White alone; Black alone, Other	4
North east; Mid west; South; West	5
Less than high school; Higher school graduate or equivalent; Some college (no degree) or associate degree; Bachelor’s degree, Advance degree	6
Past month, 2–12 months; Over a year; Never	7–9, 14–20, 44
Very curious; Somewhat curious; A little curious; Not at all	10
Definitely yes; Probably yes; Probably not; Definitely not	11–13
Strongly agree; Agree; Disagree; Strongly agree	21–26
No harm; Little harm; Some harm; A lot or harm	27–28
It will never happen; Less than a year, 1 year; 5 years; 10 years; 20 years or above	29
Strongly agree; Agree; Neither agree or disagree; Disagree; Strongly disagree	30, 38–40
Yes; No	31–37, 48–50, 54, 55, 58
Mostly A’s; A’s or B’s; Mostly B’s; B’s or C’s; Mostly C’s; C’s or D’s; Mostly D’s; D’s or F’s; Mostly F’s; School is ungraded	4
Never; Rarely; Sometimes; Often; Very often	42
Excellent; Very good; Good; Fair; Poor	43
Very easy; Somewhat easy; Somewhat difficult; Very difficult	45
None; less than 1 hour; 1 or 2 hours; 3 to 4 hours; More than 4 hours	46
Several times a day; About once a day; 3–5 times a week; 1–2 days a week; Every few weeks; Less often; Don’t have regular internet access	47
Cigarettes; Cigars; Cigarillos or filtered cigars; Smokeless or other tobacco user; No one living in the home uses tobacco	51
Married; Widowed; Divorced or separated; Never married	53
It is not allowed anywhere or at any time inside my home; It is allowed in some places or at some times inside my home; It is allowed anywhere and at any time inside my home	56, 57
Be very upset; Not be too upset; Have no reaction	59
None; Less than $1; $1 to $5; $6 to $10; $11 to $20; $21 to $50; $51 to $100; $101-$150; $151 or more	60
Never, Once, Twice; 3 or more times	61–65

Stage 1 in developing the propensity score involved separate regression analyses within each of the 11 groups. As Table 1 shows, 38 variables were eliminated from consideration at that stage, with 27 retained for stage 2, 8 considered in group A, 10 in group B and 9 in group C. Following eliminating 9 more variables at stage 2, 18 variables entered stage 3 with 6 more eliminated. After rerunning the regression analysis based on 10,671 youths with data on all 12 predictors, rather than 10,361 with data on 18 predictors, the final model was as shown in Table 2.

**Table 2.** Final model relating 12 predictor variables to ever e-cigarette use at Wave 1. The table shows the final model used for the propensity score. It is based on those 12 of the 18 predictor variables which entered stage 3 and is based on data from those 10,671 youth with data on all the 12 predictors.

Variable ^a^	Levels	N	OR (95% CI)
Age range	12–14	5738	1.000 (base)
	15–17	4933	1.949 (1.557-2.440)
Ever used alcohol at all	Yes	3441	1.000 (base)
	No	7230	0.401 (0.319-0.504)
Ever been curious about smoking a cigarette			0.639 (0.556-0.734)) ^b^
Think you will smoke a cigarette in the next year			0.486 (0.393-0.600) ^c^
Agree/disagree: Prefer friends who are	Strongly agree	716	1.000 (base)
exciting and unpredictable	Agree	2578	0.838 (0.602-1.168)
	Neither agree nor disagree	3818	0.604 (0.432-0.846)
	Disagree	2062	0.345 (0.220-0.542)
	Strongly disagree	1497	0.708 (0.439-1.143)
Reaction if parent/guardian found you	Be very upset	10255	1.000 (base)
using tobacco	Not be too upset	314	1.986 (0.383-2.853)
	Have no reaction	102	1.717 (0.798-3.693)
Gender	Male	5395	1.000 (base)
	Female	5216	0.660 (0.535-0.813)
Agree/disagree; I think I would enjoy	Strongly agree	25	1.000 (base)
using tobacco	Agree	164	6.720 (1.317-34.28)
	Disagree	2136	5.002 (1.011-24.74)
	Strongly disagree	8346	3.798 (0.760-18.98)
Agree/disagree: some products are	Strongly agree	332	1.000 (base)
safer than others	Agree	2621	0.514 (0.358-0.738)
	Neither agree nor disagree	2192	0.515 (0.347-0.764)
	Disagree	2317	0.348 (0.225-0.538)
	Strongly disagree	3209	0.354 (0.230-0.544)
Ever used prescription drug not	Yes	132	1.000 (base)
prescribed to you: Ritalin or Adderall	No	10539	0.323 (0.198-0.528)
Has a Facebook, Google Plus,	Yes	8909	1.000 (base)
MySpace, Twitter or other social networking	No	1762	0.468 (0.299-0.733)
Anyone who lives with you now use	Cigarettes, cigars,	2929	1.000 (base)
Tobacco	cigarillos, filtered cigars		
	Smokeless or other	456	1.389 (0.924-2.088)
	tobacco		
	No one living in the home	7286	0.741 (0.594-0.925)
	uses tobacco		

^a^The variables are shown in order of their inclusion into the model ^b^Odds ratio are per unit of the graded variable which represents decreasing curiosity ^c^Odds ratio are per unit of the graded variable which represents decreasing likelihood

Ever e-cigarette use was independently associated with older age, male sex, use of alcohol and prescription drugs, social networking, and preferring exciting and unpredictable friends. It was also associated with cohabitants using tobacco, parents or guardians not being very upset if they found the youth using tobacco, agreeing that some tobacco products are safer than others, the youth being curious about smoking, and the youth thinking they will smoke a cigarette in the next year. Note that, for these last two variables, the grading system ascribed lower scores for greater curiosity or greater likelihood to smoke cigarettes in the next year so the fitted ORs were <1. The results for the variable regarding enjoying using tobacco was less straightforward to interpret as very few youths strongly agreed they would enjoy it. However, those who strongly disagreed that they thought that they would enjoy using tobacco had a clearly lower odds of ever e-cigarette use than those who simply agreed or disagreed.

As Table 3 shows, the unadjusted OR for the association of vaping by Wave 1 with cigarette smoking initiation by Wave 2 was 5.702 (95% CI 4.334-7.502). The OR was markedly reduced by adjustment for the propensity score, whether as quintiles (2.476, 1.852-3.310), as a continuous variable (2.474, 1.791-3.419), or for the 12 variables making up the score (1.847, 1.347-2.533). Table 3 also shows the effects of introducing the variables successively. With one minor exception, introducing each variable reduced the OR, the largest reductions relating to the first four variables considered, which in combination already reduced the OR to 2.185 (1.608-2.969).

**Table 3.** Predicting Wave 2 ever smoking from Wave 1 ever e-cigarette use – effects of confounder adjustment. Table 3 first shows the unadjusted OR, and then the OR adjusted for the propensity score in two ways. Finally, it shows the results of a stepwise regression based on successively including the most significant adjustment variables.

Adjustment variables	OR (95% CI)
None	5.702 (4.334-7.502)
Propensity score as quintiles	2.476 (1.852-3.310)
Propensity score as continuous variable	2.474 (1.791-3.419)
Age range	4.806 (3.637-6.351)
+ Ever used alcohol at all	3.799 (2.855-5.055)
+ Ever been curious about smoking a cigarette	2.852 (2.123-3.831)
+ Think you will smoke a cigarette in the next year	2.185 (1.608-2.969)
+ Agree/disagree: Prefer friends who are exciting and unpredictable	2.111 (1.552-2.869)
+ Reaction if parent/guardian found you using tobacco	2.025 (1.489-2.756)
+ Gender	2.028 (1.489-2.761)
+ Agree/disagree; I think I would enjoy using tobacco	1.939 (1.420-2.648)
+ Agree/disagree: some products are safer than others	1.925 (1.406-2.635)
+ Ever used prescription drug not prescribed to you: Ritalin or Adderall	1.865 (1.360-2.557)
+ Has a Facebook, Google Plus, MySpace, Twitter or other social networking	1.852 (1.350-2.539)
+ Anyone who lives with you now use tobacco	1.847 (1.347-2.533)

### Sensitivity analyses

Table 4 summarizes the sensitivity analysis results, comparing them with those from the main analysis. While the number of significant variables included varies between analyses, all show that adjustment markedly reduces the unadjusted association, reducing ORs of over 5 to less than 3. The effect of adjustment was always greater when made for each of the individual variables making up the score. Relating ever vaping by Wave 1 to initiation of cigarette smoking by Wave 2 (main analysis, sensitivity analyses 1 and 2), the lowest adjusted ratio of 1.586 (1.194-2.198) is seen in sensitivity analysis 2, where adjustment is made for 13 predictor variables, including smoking of other products. Here, the adjustment explains 87.5% of the unadjusted association (as estimated from the ratio of the excess ORs, i.e. OR – 1). Sensitivity analysis 3, which concerns current (last 30 day) rather than ever use of both products also produced similar results, though the estimates are more variable due to the very few new cigarette smokers among e-cigarette users. Sensitivity analysis 4, where the score was based on variables linked to Wave 1 cigarette smoking rather than vaping also gave similar results, as did sensitivity analysis 5, where the score was based on variables linked to use of any tobacco product.

**Table 4.** Comparison of ORs (95% CIs) from main and sensitivity analysis. For the main analysis Table 4 shows the number of new smokers considered, as well as the OR (95% CI) for the relationship between ever e-cigarette use at Wave 1 and ever smoking initiation by Wave 2 with no adjustment for propensity to smoke, or with adjustment in three ways. Similar results are also shown for the five sensitivity analyses.

Analysis	Number of new smokers	Adjustment for propensity			
		None	Quintiles	Continuous	As variables
Main – relating ever e-cigarette use at Wave 1 to ever smoking initiation by Wave 2	421 (71 in e-cig users)	5.702 (4.334-7.502)	2.476 (1.852-3.310)	2.474 (1.791-3.519)	1.847 (1.347-2.533) ^a^
Sensitivity 1 – as main but exclude ever smokers of other products at Wave 1	333 (37 in e-cig users)	5.128 (3.570-7.366)	2.298 (1.577-3.349)	2.297 (1.496-3.529)	1.659 (1.098-2.508) ^b, c^
Sensitivity 2 – as main but include variable for other product use at Wave 1	421 (71 in e-cig users)	5.702 (4.334-7.502)	2.324 (1.737-3.110)	2.086 (1.487-2.925)	1.586 (1.144-2.198) ^d^
Sensitivity 3 – as main but linking current e-cigarettes at Wave 1 to current smoking at Wave 2	149 (6 in e-cig users)	6.320 (2.915-13.700)	2.860 (1.292-6.332)	2.682 (1.053-6.834)	1.741 (0.743-4.080) ^e^
Sensitivity 4 – as main but based on a propensity score for ever cigarette smoking	440 (71 in e-cig users)	5.442 (4.140-7.153)	2.286 (1.700-3.073)	2.569 (1.883-3.506)	2.096 (1.532-2.868) ^c, f^
Sensitivity 5 – as main but based on propensity score for ever any tobacco product	413 (69 in e-cig users)	5.661 (4.289-7.472)	2.430 (1.813-3.257)	2.217 (1.608-3.057)	1.822 (1.321-2.513) ^c, g^

^a^12 variables shown in Table 2

^b^10 variables (variables 1, 10, 11, 23, 32, 44, 48, 57, 59 and 62 from Table 1)

^c^Estimated using SAS version 9.4 as failed to converge using ROELEE

^d^12 variables shown in Table 2 plus ever smoked other products

^e^7 variables (variables 1, 10, 14, 19, 30 and 33 from Table 1 plus ever smoked other products)

^f^18 variables (variables 1, 6, 17, 21, 23, 24, 32, 33, 41, 42, 45, 48, 50, 51, 52, 59 and 62 from Table 1 plus ever smoked other products)

^g^16 variables (variables 1, 10, 11, 21, 24, 30, 32, 39, 41, 44, 48, 51, 59, 60, 62 and 64 from Table 1)

### Residual confounding

The propensity score used in the main analyses was revised using modified versions of the 12 predictor variables. The age range of 12–14 or 15–17 at Wave 1 was modified to be 12–13, 14, 15–16, 17 depending on whether 12–14 year-olds at Wave 1 were 15 at Wave 2, and whether 15–17 year-olds at Wave 1 were adults at Wave 2. Gender was unchanged, being consistent between waves. For three ever use variables (alcohol; prescription drugs; social networking) non-users at Wave 1 were now considered users if use was reported at Wave 2. For four variables where questions were asked at both waves (reaction if parent or guardian found using tobacco; think would enjoy tobacco; relative safety of tobacco products; cohabitant uses tobacco) the level most associated with vaping use was used (i.e. maximum for reaction and minimum for the other three). These questions were only asked of youths, so if the participant became an adult at Wave 2, the Wave 1 response was used. For the other three variables (curiosity about smoking; think will smoke a cigarette; prefer exciting and unpredictable friends) a corresponding question was not asked at Wave 2, so the Wave 1 value was used.

As the subjects included in this analysis were as for the main analysis, the unadjusted OR remained 5.702 (95% CI 4.334-7.502). After adjusting for propensity score as quintiles, the OR value reduced to 2.390 (1.791-3.188), somewhat lower than the 2.476 (1.852-3.310) in the main analysis, with no misclassification adjustment. After adjusting as a continuous variable, the OR reduced to 2.262 (1.625-3.150), again somewhat lower than the 2.474 (1.791-3.519) in the main analysis. Adjusting for the 12 variables making up the score reduced the OR to 1.772 (1.264-2.484), somewhat lower than the 1.847 (1.347-2.533) in the main analysis.

## Discussion

We have described analyses aimed at gaining further insight into the magnitude of any true “gateway effect” by attempting to control better for confounding factors linked to initiation of smoking. We used a propensity score approach, which is intended to remove confounding in the analysis of outcomes by balancing exposure groups on potential confounders, the score being developed prior to, so independently of, the analysis of outcomes. This approach attempts to transpose observational data into what would have been obtained from a randomized trial, using groups balanced on baseline covariates. The main analysis, which aims to balance potential confounders across vapers and non-vapers at Wave 1 (the comparison groups in the analysis where cigarette smoking is the outcome) is strictly designed for this approach. An alternative approach addressed using sensitivity analysis 5, views the propensity outcome more broadly, considering use of any nicotine-containing product as indicative of an inclination to initiate cigarette smoking. The difficulty in the propensity score approach, as with use of observational data generally, is to ensure that all relevant variables are considered in the score, and to account for possible inaccuracies in the variables included.

The main and five sensitivity analyses summarized in Table 4 all show that adjustment for propensity determined at Wave 1 markedly weakens the gateway effect, the association between vaping by Wave 1 and subsequent initiation of smoking. This was true whether propensity was based on variables associated with vaping (the main analyses), cigarette smoking (sensitivity analysis 4) or any tobacco product use (sensitivity analysis 5). Sensitivity analyses 1–3 also demonstrated this marked reduction, whether users of other products at Wave 1 were excluded or included, whether or not adjustment was made for such use, or whether analyses were based on ever or current use. There was no consistent difference between results adjusted for propensity as quintiles or as a continuous variable, but adjustment for the individual variables making up the score produced lower adjusted ORs. The proportion of the unadjusted excess OR (i.e. OR – 1) explained by adjustment for the individual variables was at least 75.4% in the main and sensitivity analyses, with a maximum of 87.5% for sensitivity analysis 2, where other product use was adjusted for, as well as the 12 main analysis predictor variables.

Our analyses have limitations. One is the small numbers of new smokers considered, never exceeding 71 and as low as six in sensitivity analysis 3. We are currently conducting additional work to try to obtain more precise answers by also using Wave 3 data.

A second issue is the possibility of over-adjustment. One can argue that vaping by Wave 1 may have affected some answers given then. For example, taking up e-cigarettes may make youths more curious about cigarettes, or more likely to think they will smoke or enjoy them. Using Wave 3 data, we are also conducting further analyses relating initiation of cigarette smoking at Wave 3 to vaping at Wave 2, restricting attention to those who, at Wave 1, had never vaped, and using propensity indicators recorded at Wave 1.

It is possible that more complete propensity adjustment might have explained more of the “gateway effect”. Our analyses only included variables showing an effect significant at p<0.01. Weakening significance to p<0.05 or p≤0.1 might have included more variables in the propensity score and explained even more of the association.

As is well documented, inaccurately determining confounding variables may limit the ability to fully adjust. Thus, many years ago ( Tzonou *et al*., 1986), it was demonstrated that “even misclassification rates as low as 10% can prevent adequate control of confounding” and other publications highlight the residual confounding problem ( Ahlbom & Steineck, 1992; Fewell *et al*., 2007; Greenland, 1980; Phillips & Davey Smith, 1994; Savitz & Baron, 1989). Proper adjustment for residual confounding requires a gold standard to compare reported answers with, but such data were unavailable in the PATH study. However, some insight was given by using predictor variables derived from answers given at both Wave 1 and Wave 2. Thus, if a youth reported alcohol use at one wave and not the other, it is possible this was not reported at one wave, and a predictor based on ever reported use may better predict smoking initiation. Such analyses usually weakened the adjusted association of prior vaping with subsequent smoking initiation, but only slightly. This may, however, reflect methodological limitations rather than lack of serious residual confounding.

While our analyses make it clear that most of the observed relationship between vaping and subsequent initiation of smoking results from confounding, the significant association seen even after extensive adjustment for confounders does seem to be consistent with there being some true gateway effect. However concerns about incomplete adjustment for confounding remain and our results do not unequivocally demonstrate that any true effect exists. More reliable information emerging from the further analyses we are currently conducting using Wave 3, should provide a better insight into the magnitude of any true gateway effect.

Another gateway analysis based on PATH Waves 1 and 2 has recently been published ( Watkins *et al*., 2018). This differed from ours in that their “unadjusted” model already included all non-cigarette tobacco products as indicators of cigarette initiation, and their “adjusted” models added only a restricted list of predefined variables, rather than using models to include more relevant variables. While their adjusted models did include some established determinants of cigarette use (sensation seeking; alcohol use; living with a tobacco user; and variables regarding health warnings and advertising), their adjusted ORs were higher than ours. Thus, whereas the variables we included in our main analysis reduced the OR from 5.702 to 1.847, their similar analysis reduced it only from 3.50 to 2.53. Consequently, although they recognized uncontrolled confounding may exist, they dubiously considered vaping was “independently associated with cigarette smoking one year later”.

In considering whether a true important gateway effect exists, one should note the lack of any increase in the US in cigarette smoking prevalence following the rise in vaping ( Levy *et al*., 2019), and the fact that, in the PATH study, considerably more (279 vs. 79) Wave 1 cigarette only smokers took up e-cigarettes by Wave 2, than Wave 1 e-cigarette only users who took up smoking. Despite any possible gateway effect, introducing e-cigarettes may have reduced overall youth smoking prevalence.

## Conclusions

The results presented, based on Waves 1 and 2, strongly suggest that reported estimates of the gateway effect ( Soneji *et al*., 2017) are much too high. Indeed, it is not completely clear whether vaping actually increases subsequent uptake of cigarette smoking if potential confounding effects were to be fully accounted for.

## Addendum

At the time this paper was being finalised, an analysis was published ( Berry *et al*., 2019a) investigating gateway effects based on data from Waves 1, 2 and 3 of the PATH study. The authors reported that prior e-cigarette use was associated with increases in the odds of ever and current cigarette use by, respectively, 4.09 (95% CI 2.97-5.63) and 2.75 (1.60-4.73). Though noting that they could not rule out the possibility of residual confounding, they concluded that their findings supported a gateway effect. We will examine this claim in detail based on the results of our ongoing analyses using the data from all three Waves.

## Data availability

### Underlying data

National Addiction & HIV Data Archive Program: Population Assessment of Tobacco and Health (PATH) Study [United States] Public-Use Files (ICPSR 36498). https://doi.org/10.3886/ICPSR36498.v8 ( United States Department of Health and Human Services, 2018).

Data are available under the Terms of Use as set out by ICPSR, which can be accessed when users start the process of downloading the data.

### Extended data

Open Science Framework: Investigating gateway effects using the PATH study https://doi.org/10.17605/OSF.IO/QCFZR ( Lee, 2019).

This project contains the following extended data files:

- Gateway PATH_F1000 Research_Supplementary file.docx

Data are available under the terms of the Creative Commons Zero “No rights reserved” data waiver (CC0 1.0 Public domain dedication).
