# Supplementary material for: Investigating gateway effects using the PATH study
Source: F1000Res. 2019 Dec 4;8:264. Originally published 2019 Mar 7. [Version 2] doi: 10.12688/f1000research.18354.2 (PMC6950312; doi:10.12688/f1000research.18354.2)
Supplement: Peer review history

# Peer review: Reviewer response for version 2

**DOI:** 10.5256/f1000research.23710.r57453

**Published:** 2020-01-08

**Authors:** Reiner Hanewinkel

**Affiliations:** 1 Institute for Therapy and Health Research (IFT-Nord), Kiel, Germany

I do not fully agree with the arguments raised by the authors of the paper, but that's normal in science.

 The data are presented in a way, that every reader can make up his own mind about the conclusions made by the authors. For that reason, I approve the paper.

I confirm that I have read this submission and believe that I have an appropriate level of expertise to confirm that it is of an acceptable scientific standard.

# Peer review: Reviewer response for version 1

**DOI:** 10.5256/f1000research.20078.r55826

**Published:** 2019-11-08

**Authors:** Reiner Hanewinkel

**Affiliations:** 1 Institute for Therapy and Health Research (IFT-Nord), Kiel, Germany

“On 19 December 2018, Altria announced an offer of US$12.8billion for a 35% share of Juul Labs. The deal combines Altria (formerly Philip Morris), the company with the largest share of the US cigarette market, with the large and rapidly growing vaping product (aka e-cigarette) company, Juul Labs. The acquisition price was based on a US$38billion valuation, which was more than twice Juul Labs’ August valuation, and a surprise to many investors on Wall Street.” ^1^

 We have to have this information in mind, because the manuscript submitted to F1000Research by Peter Lee and John Fry is financed by Philip Morris. My personal opinion is that Philip Morris has an interest to show that the so called gateway effect is not existent.

 I am not an expert in the propensity score approach, but what I understand is, that the authors tried everything to show, that the gateway effect does not exist. It seems trivial to me, that adjustments reduce an OR. Even after adjustment for a broad range of confounders, there is still a significant and clinical meaningful association between e-cigarette use at baseline and smoking initiation in the observational period.

 My main point is that I do not agree with the conclusion and discussion of the results. Instead of saying that confounding is a major factor explaining most of the observed gateway effect, the authors should emphasize that even after controlling for a large number of confounders, the gateway effect is still present.

 I am counting nearly two dozens of cohort studies, which investigated the gateway effect. The authors only cite a portion of these studies. In detail, young people have been recruited in the US and Canada ^2^^-^^15^, UK ^16^^-^^18^, Finland ^19^, Mexico ^20^, the Netherlands ^21^, Romania ^22^, Taiwan ^23^, and Germany ^24^. More than 100,000 young people have been recruited for these studies with observational periods of up to 24 months. Outcome variables range from one cigarette to daily smoking. All these studies found a significant association between e-cigarette use at baseline and uptake of smoking in the observational period. Applying the well-known Bradford-Hill criteria, it seems to me that the following criteria are fulfilled: (1) Strength (effect size), (2) Consistency (reproducibility), (3) Temporality, and (4) Plausibility.

 In the Introduction the authors should describe the two opponent explanations for the observed association between e-cigarette use and the later uptake of smoking: the so called “common liability” theory vs. the “gateway” theory ^25^. Another important theoretical paper should also be discussed ^26^.

 If anything, the results reported are in line with the gateway theory and not with the common liability theory.

I confirm that I have read this submission and believe that I have an appropriate level of expertise to state that I do not consider it to be of an acceptable scientific standard, for reasons outlined above.

## References

[1] : Altria-Juul Labs deal: why did it occur and what does it mean for the US nicotine delivery product market. *Tob Control*.2019; 10.1136/tobaccocontrol-2019-055081 10.1136/tobaccocontrol-2019-055081 31484802PMC7279136

[2] : E-Cigarettes and Future Cigarette Use. *Pediatrics*.138(1) : 10.1542/peds.2016-0379 10.1542/peds.2016-0379 27296866PMC4925085

[3] : Association of Electronic Cigarette Use With Initiation of Combustible Tobacco Product Smoking in Early Adolescence. *JAMA*.2015;314(7) : 10.1001/jama.2015.8950 700-7 10.1001/jama.2015.8950 26284721PMC4771179

[4] : Exclusive e-cigarette use predicts cigarette initiation among college students. *Addict Behav*.2018;76: 10.1016/j.addbeh.2017.08.023 343-347 10.1016/j.addbeh.2017.08.023 28892771PMC5614895

[5] : E-cigarette use as a predictor of cigarette smoking: results from a 1-year follow-up of a national sample of 12th grade students. *Tob Control*.26(e2) : 10.1136/tobaccocontrol-2016-053291 e106-e111 10.1136/tobaccocontrol-2016-053291 28167683PMC5545162

[6] : Electronic cigarette use and uptake of cigarette smoking: A longitudinal examination of U.S. college students. *Addict Behav*.67: 10.1016/j.addbeh.2016.12.009 66-72 10.1016/j.addbeh.2016.12.009 28038364PMC5250543

[7] : E-cigarette use and subsequent cigarette and marijuana use among Hispanic young adults. *Drug Alcohol Depend*.2016;163: 10.1016/j.drugalcdep.2016.04.027 261-4 10.1016/j.drugalcdep.2016.04.027 27141841PMC7453602

[8] : Longitudinal study of e-cigarette use and onset of cigarette smoking among high school students in Hawaii. *Tob Control*.26(1) : 10.1136/tobaccocontrol-2015-052705 34-39 10.1136/tobaccocontrol-2015-052705 26811353PMC4959970

[9] : Association of e-Cigarette Vaping and Progression to Heavier Patterns of Cigarette Smoking. *JAMA*.2016;316(18) : 10.1001/jama.2016.14649 10.1001/jama.2016.14649 PMC558082027825000

[10] : Electronic cigarette use and smoking initiation among youth: a longitudinal cohort study. *CMAJ*.2017;189(43) : 10.1503/cmaj.161002 E1328-E1336 10.1503/cmaj.161002 29084759PMC5662449

[11] : Trajectories of E-Cigarette and Conventional Cigarette Use Among Youth. *Pediatrics*.141(1) : 10.1542/peds.2017-1832 10.1542/peds.2017-1832 29203523PMC5744268

[12] : Initiation of Traditional Cigarette Smoking after Electronic Cigarette Use Among Tobacco-Naïve US Young Adults. *Am J Med*.131(4) : 10.1016/j.amjmed.2017.11.005 443.e1-443.e9 10.1016/j.amjmed.2017.11.005 29242110PMC7054856

[13] : Progression to Traditional Cigarette Smoking After Electronic Cigarette Use Among US Adolescents and Young Adults. *JAMA Pediatr*.2015;169(11) : 10.1001/jamapediatrics.2015.1742 1018-23 10.1001/jamapediatrics.2015.1742 26348249PMC4800740

[14] : Association of Noncigarette Tobacco Product Use With Future Cigarette Smoking Among Youth in the Population Assessment of Tobacco and Health (PATH) Study, 2013-2015. *JAMA Pediatrics*.2018;172(2) : 10.1001/jamapediatrics.2017.4173 10.1001/jamapediatrics.2017.4173 29297010PMC5801043

[15] : E-Cigarette Use and Future Cigarette Initiation Among Never Smokers and Relapse Among Former Smokers in the PATH Study. *Public Health Rep*.134(5) : 10.1177/0033354919864369 528-536 10.1177/0033354919864369 31419184PMC6852065

[16] : Relationship between trying an electronic cigarette and subsequent cigarette experimentation in Scottish adolescents: a cohort study. *Tob Control*.2017; 10.1136/tobaccocontrol-2017-053691 10.1136/tobaccocontrol-2017-053691 28735273PMC6047138

[17] : Do electronic cigarettes increase cigarette smoking in UK adolescents? Evidence from a 12-month prospective study. *Tob Control*.2017; 10.1136/tobaccocontrol-2016-053539 10.1136/tobaccocontrol-2016-053539 28818839PMC6047139

[18] : The Association Between Smoking and Electronic Cigarette Use in a Cohort of Young People. *J Adolesc Health*.62(5) : 10.1016/j.jadohealth.2017.11.301 539-547 10.1016/j.jadohealth.2017.11.301 29499983PMC5938086

[19] : Nicotine matters in predicting subsequent smoking after e-cigarette experimentation: A longitudinal study among Finnish adolescents. *Drug Alcohol Depend*.2019;201: 10.1016/j.drugalcdep.2019.04.019 182-187 10.1016/j.drugalcdep.2019.04.019 31238240

[20] : A longitudinal study of electronic cigarette use and onset of conventional cigarette smoking and marijuana use among Mexican adolescents. *Drug and Alcohol Dependence*.2017;180: 10.1016/j.drugalcdep.2017.09.001 427-430 10.1016/j.drugalcdep.2017.09.001 PMC577144028988005

[21] : E-cigarette and waterpipe use in two adolescent cohorts: cross-sectional and longitudinal associations with conventional cigarette smoking. *Eur J Epidemiol*.33(3) : 10.1007/s10654-017-0345-9 323-334 10.1007/s10654-017-0345-9 29260431PMC5889768

[22] : Bidirectional associations of e-cigarette, conventional cigarette and waterpipe experimentation among adolescents: A cross-lagged model. *Addict Behav*.80: 10.1016/j.addbeh.2018.01.010 59-64 10.1016/j.addbeh.2018.01.010 29355818PMC5807159

[23] : Electronic Cigarette Use and Smoking Initiation in Taiwan: Evidence from the First Prospective Study in Asia. *International Journal of Environmental Research and Public Health*.2019;16(7) : 10.3390/ijerph16071145 10.3390/ijerph16071145 PMC648059530935027

[24] : E-Cigarettes and the Use of Conventional Cigarettes. *Deutsches Aerzteblatt Online*.2018; 10.3238/arztebl.2018.0243 10.3238/arztebl.2018.0243 PMC593854729716689

[25] : Gateway effects and electronic cigarettes. *Addiction*.113(10) : 10.1111/add.13924 1776-1783 10.1111/add.13924 28786147

[26] : Vaping as a Catalyst for Smoking? An Initial Model on the Initiation of Electronic Cigarette Use and the Transition to Tobacco Smoking Among Adolescents. *Nicotine Tob Res*.2016;18(5) : 10.1093/ntr/ntv193 647-53 10.1093/ntr/ntv193 26386472

# Response

**Published:** 2019-11-27

**Authors:** Peter Lee

**Affiliations:** P.N.Lee Statistics and Computing Ltd, UK

We thank Dr. Hanewinkel for his comments. However, it seems unfortunate that he suggests our interpretation may have been affected by the funding source. As experienced medical statisticians/epidemiologists our interest is in unbiased assessment of data, not in trying to reach conclusions favourable to any sponsor. The objective of our paper was to provide insight into how much the observed gateway effect could be explained by adjustment for confounding factors, in order to provide insight into the possible magnitude of any true effect. That adjustment could reduce an unadjusted OR from 5.70 to 1.59 should be interpreted as providing doubt as to whether there is any real effect, despite the 1.59 being statistically significant. Our discussion, and also our companion paper in F1000Research ^[1]^, already highlight the potential problems of interpretation due to residual confounding.

Dr. Hanewinkel states that “it seems trivial” to him “that adjustments reduce an OR”. However, theoretically adjustment can increase or decrease an OR. He cites numerous cohort studies that reported a significant gateway effect after adjustment, and we have cited all but the most recently published ones in our present paper or in its companion ^[1]^. We note that many of these papers have also shown a large reduction in the OR after allowing for confounding variables. The existence of a significant association following adjustment does not necessarily mean that the adjusted association is real, for the reasons we have already discussed. 

We have now extended the introduction as suggested to describe the “common liability” and the “gateway” theory, making it clear that both can contribute to the observed association between e-cigarette use and subsequent initiation of smoking.

Our abstract already made clear that a significant association remained after adjustment, and that its interpretation is not straightforward. We have now modified the conclusion in the abstract to point out that further work is needed to clarify the extent of any true gateway effect that may exist.

We have also extended the discussion section of the paper. We feel that our results adequately support our conclusion that “confounding is a major factor, explaining most [though not all] of the observed gateway effect”. Further analysis of later waves of the PATH study could well help to establish whether the residual effect is or is not real.

Reference

1          Lee PN, Coombs KJ and Afolalu EF. Considerations related to vaping as a possible gateway into cigarette smoking: an analytical review [version 3; peer review: 2 approved]. *F1000Research* 2018; **7**: ( https://doi.org/10.12688/f1000research.16928.3) [DOI: doi.org/10.12688/f1000research.16928.3]

# Peer review: Reviewer response for version 1

**DOI:** 10.5256/f1000research.20078.r48433

**Published:** 2019-06-12

**Authors:** Raymond Niaura, Shu Xu

**Affiliations:** 1 Department of Social and Behavioral Sciences, NYU College of Global Public Health, New York City, NY, USA; 2 Department of Biostatistics, NYU College of Global Public Health, New York City, NY, USA

The authors aimed to evaluate the “gateway effect” of baseline e-cigarette use predicting cigarette smoking initiation among youths, addressing the inadequate adjustment of confounders considered in the previous published studies. The first two waves of PATH data from the youth sample were used for this purpose. 

 The strength of this study is that the authors used multiple approaches to evaluate this gateway effect, and performed five sets of sensitivity analysis. Propensity scores for e-cigarette use at wave 1 were created. Propensity score based approaches are preferred to statistically control for numerous confounding variables; however, this approach was not completely adequate as it appears in this study for several reasons. A few major concerns are below.

 First, due to the availability of data at the time of analyses, the confounding variables were measured concurrently with e-cigarette exposure or afterwards (i.e., analyses investigating residual confounding). Considering the Wave 3 PATH data have been available since fall 2018, the authors are encouraged to revise the manuscript using data from the first 3 waves. They state they will do so but they could probably access the data at this point.

 Second, the authors aimed to address the inadequate adjustment of confounding in previous studies, however, this limitation still exists in the current study. As a result of Step 1, only a set of 13 covariates were included of the main analysis, sensitivity analyses, and residual confounding analysis. Moreover, it is unclear whether the balancing of covariates was achieved between the (e-cigarette) exposure and non-exposure groups. 

 A few details regarding each approach need to be provided. Specifically, it was unclear what the authors meant by saying “the first analysis was a weighted logistic regression analysis.” How was a weighting variable created? Was this what the authors called “no adjustment of propensity” afterwards (in the first paragraph of the Sensitivity Analysis section, and in Table 4)? The results from the “adjustment as quintiles” and “adjustment as a continuous variable” approaches typically agree, and the effect of e-cigarette exposure would be biased if only a subset of confounding variables were considered in analyses. Moreover, the “using the variables making up the score individually” approach was actually a traditional covariate approach, which assumed a linear association between the outcome and each covariate. Authors need to justify whether this assumption was satisfied or not before they concluded the findings of the study.

 Third, in analyses investigating residual confounding, authors used some of the covariates from Wave 2, which might be influenced by the exposure of “e-cigarette” at Wave 1. In this case, the partial coefficient of e-cigarette use on outcome is actually the controlled direct effect in a mediation analysis. This coefficient only showed the partial effect of e-cigarette use on outcome, which was an underestimated total effect of exposure. In this set of analysis, the confounding was over-adjusted.

 Fourth, all analyses only involved participants with complete data for each variable considered in the group being analyzed. Missing complete at random was actually assumed in all analyses. Authors need to report the sample sizes of the original data and the working data, and justify whether/why this assumption was valid in this study.

 In addition, a few minor issues are below.

1. Methods: Some information in the last paragraph of the Introduction section need to be relocated in the Methods section, under the title of “sample and data collection”.
2. Software: what is the ROELEE program? Please briefly introduce this software. Under what circumstance did ROELEE fail to converge (and SAS did not)? 
3. Supplementary document: the very long list of variables was not helpful to understand how confounding variables were selected. It seems like the authors have already selected (without details on criteria) 60 smoking predictor variables out of 200+. Please provide sample questions as needed.
4. Sensitivity analysis: report sample size of each sensitivity analysis in Table 4.

We confirm that we have read this submission and believe that we have an appropriate level of expertise to confirm that it is of an acceptable scientific standard.

# Response

**Published:** 2019-06-27

**Authors:** Peter Lee

**Affiliations:** P.N.Lee Statistics and Computing Ltd, UK

Response by the first author, Peter Lee.

In his first major point, Dr Niaura expressed concern about the possibility of confounding arising if vaping by Wave 1 had affected some answers given then. We noted this in our discussion saying that further analyses are planned relating initiation of cigarette smoking at Wave 3 to vaping at Wave 2, restricting attention to those who, at Wave 1, had never vaped, and using propensity indicators recorded at Wave 1. We have had access to the Wave 3 data for some time, and in due course will publish a further paper. We do not propose to revise the current manuscript.

As regards to the second concern, our final analyses were based on adjustment for a limited number of confounding variables. However, this list was based on a much longer list, with no further variables adding significantly to the final model, the much longer list (shown in Supplementary File 1) being itself derived from a review of the literature. The fact that the model enormously reduced the association between vaping and subsequent initiation of cigarette smoking was the main point we wished to make. It is possible other models may explain more of the unadjusted association but that will be addressed in the paper currently being prepared.

Dr. Niuara refers to reporting whether the balancing of covariates was achieved between the e-cigarette exposed and non-exposed groups. Presumably, he is referring to balancing after propensity adjustment. We intend to report on this in the planned new paper. 

Dr. Niaura asked how the weighting variable was created. This variable is supplied directly to users of the PATH study and allows the user to generate results representative of the U.S. population. 

It is stated that “the effect of e-cigarette exposure would be biased if only a subset of confounding variables were considered in analyses”. As noted above we did take into account a very large number of potential confounders in our analyses. It would be expected that adjustment for some variables would not in fact have a material effect when other variables were already included in the analyses, i.e. they would not be true confounders. 

As regards linear associations it should be noted that most of the variables were yes/no variables. We will consider non-linear relationships in our planned analyses using Wave 3.

It is well known that adjustment for inaccurately measured confounding variables may be incomplete. In the absence of a gold standard we attempted to gain insight into the problem of looking at variation in answers to corresponding questions at different Waves. It is true that this may cause problems if the answers are affected by e-cigarette use, but this could only be addressed by analyses involving more than three Waves, relating e-cigarette use at Wave 3 to initiation of smoking by Wave 4, with adjustment for questions asked at Waves 1 and 2 in non-tobacco users.

We will report fuller details regarding missing data in our planned analyses based on Waves 1 to 3.

As regards the other points we do not propose to modify the current paper, preferring to take all the points into account in the paper on Waves 1 to 3. 

For information, the ROELEE program is one my company started to develop over 30 years ago, initially to get output in a user-friendly form not available using programs like SAS or SPSS. It has undergone numerous updates over the years and is fully tested. It is available from ROELEE Statistics Ltd. While, for logistic regression analyses, it gives the same results as SAS, but in a more convenient form, the version of ROELEE we used at the time sometimes failed to converge when there are large numbers of predictor variables, whereas SAS did not. We are currently modifying the code of ROELEE to avoid this non-convergence problem.

As regards the sample sizes used in the 6 sets of analyses in Table 4, these were as follows:

Main, S2 and S5 9423; S1 8900; S3 9907; S4 9354.
